# Field evaluation in Chad of community usage of CD4 T lymphocyte counting by alternative single-platform flow cytometry

**DOI:** 10.1186/1472-6963-13-373

**Published:** 2013-10-01

**Authors:** Donato Koyalta, Mohammad-Ali Jenabian, Barou Djouater, Noël Djemadji-Oudjeil, Francois-Xavier Mbopi-Keou, Angélique Ndjoyi-Mbiguino, Laurent Bélec

**Affiliations:** 1 Ministère de la Santé Publique, BP 407 N’Djamena, Chad; 2 Chronic Viral Illness Service and Research Institute, McGill University Health Centre, Montreal, Canada; 3 Conseil National de Lutte contre le SIDA, Primature, N’Djamena, Chad; 4 Organisation Mondiale de la Santé, Représentation du Tchad, N’Djamena, Chad; 5 National Public Health Laboratory, Ministry of Public Health, University of Yaounde I, Yaounde, Cameroon; 6 Laboratoire National de Référence des Maladies Sexuellement Transmissibles et du SIDA, Département de Microbiologie, Faculté de Médecine de Libreville, Université des Sciences de la Santé, Libreville, Gabon; 7 Laboratoire de virologie, hôpital Européen Georges Pompidou, and Faculté de Médecine Paris Descartes, Université Paris Descartes (Paris V), Sorbonne Paris Cité, Paris, France

**Keywords:** Flow cytometry, CD4 T cell count, CD45, Decentralization, Sub-Saharan Africa, Chad

## Abstract

**Background:**

Field and community evaluation of the routine usage of CD4 T counting platforms is essential in resource-poor countries for efficient and cost-effective monitoring of HIV-infected adults and children attending health care centers.

**Methods:**

We herein addressed the principal issues raised by the implementation of the single-platform, volumetric Auto40 flow cytometer (Apogee Flow Systems Ltd, Hemel Hempstead, UK) in 8 community HIV monitoring laboratories of different levels throughout Chad. This is a country with particularly difficult conditions, both in terms of climate and vast geographical territory, making the decentralization of the therapeutic management of HIV-infected patients challenging.

**Results:**

The routine usage of the Auto40 flow cytometers for a period of 5 years (2008–2013) confirms the reliability and robustness of the analyzer for community-based CD4 T cell enumeration in terms of both absolute numbers and percentages to enable accurate monitoring of HIV-infected adults and children. However, our observations suggest that the Auto40 mini flow cytometer is not suitable for all laboratories as it is oversized and ultimately very expensive.

**Conclusion:**

The Chad experience with the Auto40 flow cytometer suggests that its usage in resource-limited settings should be mainly reserved to reference (level 1) or district (level 2) laboratories, rather than to laboratories of health care centres (level 3).

## Background

The HIV epidemic remains a major global public health challenge with a total of 34.0 million people living with HIV worldwide [1]. During the past decade, there has been a remarkable global effort to improve access to HIV antiretroviral therapy (ART). Despite this progress, approximately half of all people who need treatment are not yet receiving it. The need for ART is most critical in sub-Saharan Africa, where less than 40% of eligible patients currently receive treatment [1,2]. The guidelines of the World Health Organization (WHO) for the scaling up of ART in adults and children living in resource-limited settings [3-5] emphasize the necessity of laboratory monitoring which is initially based on immunological assessment by the enumeration of CD4 T lymphocytes. This enumeration helps guide timing of ART initiation and assists in monitoring immunological responses in patients on ART. In addition, measurement of the HIV-1 RNA load is now recommended in order to monitor virological responses, identify early therapeutic failures and assess virological outcomes following therapeutic switches [6,7].

Affordable CD4 T cell enumeration has gradually become possible through the use of simple, compact and robust low-cost new generation, point-of-care flow cytometers. These devices operate as single-platform volumetric instruments without the use of expensive microbeads [8-10]. Introduced in 2005, the recently developed Auto40 flow cytometer (Apogee Flow Systems Ltd, Hemel Hempstead, UK; http://www.ApogeeFlow.com) was originally designed for military applications [11]. The Auto40 assay is based on a no-lyse procedure [12] which avoids the need for red blood cell lysis. This, in turn, reduces assay variability due to changes in assay conditions, such as time and temperature of incubation, as well as differences in the effects of lysis reagents on cells following exposure [13]. The Auto40 analyzer uses a volumetric syringe powered by a stepper motor that draws and delivers a known sample volume. Therefore, the absolute volumetric counting allows the direct determination of the number of cells per unit of sample volume without the need for reference material such as microbeads [8,14]. The Auto40 analyzer was initially intended for CD4 T cell enumeration based on primary CD4 gating and has been validated in Senegal for measurement of absolute CD4 T cell count by reference to the FACSCount system [11]. The analyzer has been recently updated for CD4 T cell measurement using a protocol involving primary CD45 gating followed by secondary CD4 gating. The CD45-assisted method for CD4 T cells enumeration in percentage has been previously demonstrated to allow accurate CD4 T cell counting, when used in dual [15-17] as well as single-platforms, either volumetric [18] or bead-based [19,20]. The updated version of the Auto40 analyzer has been recently validated for the enumeration of both absolute numbers and percentages of CD4 T cells in Cameroon [21,22] as well as in Chad [23] and is therefore addressing the current WHO recommendations for CD4 T cell measurements in children less than 5 years [5].

The objective of the present study was to assess the performance of the Auto40 CD45-assisted flow cytometer at the scale of a sub-Saharan African country whereby there are harsh climatic conditions and challenges in delivering operational public health programs. Indeed, the conditions within many community laboratories, where several Auto40 analyzers have been implemented, are not similar to the conditions within the laboratories in large cities and academic medical centers where the Auto40 flow cytometer has been previously evaluated. Such evaluations have occurred in laboratories in Dakar, Senegal [11], Yaoundé, Cameroon [21,22] and Chad [23]. Furthermore, Chad is a country with particularly difficult conditions in terms of both climate and bad infrastructures making the decentralization of the therapeutic management of HIV-infected patients especially challenging. Therefore, in this study, we addressed the principal issues raised by 4 years of routine usage of the Auto40 flow cytometers implemented in 8 HIV monitoring laboratories of different levels throughout Chad.

## Methods

### Auto40 flow cytometers installed in Chad

The Auto40 flow cytometers (Apogee Flow Systems Ltd) installed in Chad are equipped with a green laser at 532 nm, a side scatter detector, two fluorescence channels and means for direct volumetric counting which is does not require the lysis of red blood cells. In brief, direct volumetric CD4 T cell measurements are performed on the Auto40 using phycoerythrin (PE)-conjugated anti-CD4 and PE-Dyomics649-conjugated anti-CD45 monoclonal antibodies (Apogee Flow Systems Ltd). The Auto40 analytical procedure avoids the need for a cell washing step. Thus, 50 μl of whole EDTA-blood is added into polypropylene test tubes containing pre-dispensed, stabilized monoclonal antibodies. After 25 minutes of incubation at room temperature in the dark, the blood samples are diluted 1:10 in phosphate buffered saline. The non-lysed-non-washed stained samples are run on the Auto40 flow cytometer and the CD4 T cell counts are obtained in absolute numbers and in percentages.

**Figure 1:**
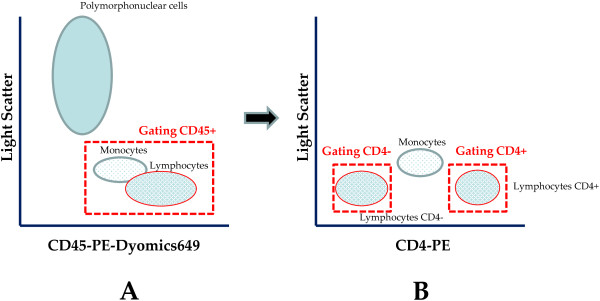
**Gating strategy for the volumetric Auto40 flow cytometer (Apogee Flow Systems Ltd, Hemel Hempstead, UK), as depicted from typical figure provided by “Auto-Lymphocyte” software (Apogee Flow Systems Ltd) for the counting of CD4 T lymphocytes in one K**_**3**_**-EDTA-anticoagulated blood sample, incubated with phycoerythrin (PE)-conjugated anti-CD4 and PE-Dyomics649-conjugated anti-CD45 monoclonal antibodies (Apogee Flow Systems Ltd): (A) CD45-PE-Dyomics649 *× *axis-light scatter dot plot scattergram; the primary CD45 gating differentiates clearly the CD45-positive lymphocytes and monocyte populations, and the population of polymorphonuclear cells; (B) CD4-PE *× *axis-light scatter dot plot scattergram; double secondary CD4 gating allows counting independently the population of CD4-negative lymphocyte and CD4-positive lymphocytes; the CD4 T lymphocytes count in absolute number is given by the CD4-positive gating; the CD4 T lymphocytes count in percentage corresponds to the ratio of CD4-positive lymphocytes count in absolute number to the total CD4-postive and CD4-negative lymphocytes counts, corresponding to the sum of both secondary gating.**

The simplified protocol used by the Auto40 flow cytometer is based on a sequential strategy initially involving gating on the total white blood cell population (CD45-positive population) which serves as the common denominator to enumerate CD4-positive T lymphocytes, instead of primarily gating on the lymphocyte population, a method which is error-prone [24]. The specific use of the side scatter parameter allows for the discrimination of monocytes due to the high side scatter and low CD4 expression of monocytes. This enables for the accurate enumeration of CD4-positive T cells as they display, in contrast, to monocytes, low side scatter and high CD4 expression [15]. The analysis on the Auto40 flow cytometer is automatically performed by the built-in software “Auto-lymphocyte” (Apogee Flow Systems Ltd), with the option of controlling and assessing the quality of the data analysis. CD45-positive lymphocytes and monocytes are identified by primary gating on bright CD45 fluorescent cells in a CD45xside scatter dot plot scattergram (Figure 1A). The CD45 fluorescent polymorphonuclear cells are excluded from the gating according to their high nuclear density. Within the CD45-positive cells, CD4-positive and CD4-negative lymphocytes are identified by secondary gatings in a CD4xside scatter dot plot scattergram (Figure 1B). CD4-positive T lymphocytes are easily separated from monocytes and CD4-negative lymphocytes, and counted independently. The CD4 T lymphocyte count in absolute number corresponds to the CD4-positive secondary CD4 gating cell populations. The CD4 T lymphocytes count in percentage corresponds to the ratio of CD4-positive lymphocytes count in absolute number to the total CD4-positive and CD4-negative lymphocytes counts, corresponding to the sum of both secondary CD4 gatings. The sample lecture takes around 2 minutes. Aside from simplifying the sample preparation, the software “Auto-lymphocyte” allows automatic lecture which greatly reduces manipulations required by laboratory technicians. The entire procedure is rapid and only requires 30 minutes to complete. According to the manufacturer, the Auto40 flow cytometer has the theoretical capacity to perform 150 CD4 T cell enumerations per day.

Quality control on the Auto40 flow cytometer implemented in Chad is assessed by the use of a calibrated bead sample (Apogee cat # 1444 for Auto40-green, Apogee Flow Systems Ltd) at the beginning and end of each session, and on the use of stabilized blood reference samples (Cytofix CD4, CaltagMedsystems Ltd, UK) as an additional external control.

The Senegalese validation used the initial Auto40 version based on primary CD4 gating and focused exclusively on CD4 T cell counting in absolute numbers [11]. However, as the percentage of CD4 T cells is essential for monitoring HIV disease progression in children less than 5 years [3,10], the primary CD4 gating format of the Auto40 analyzer that only measures absolute CD4 T cell numbers was modified by the manufacturer in 2006 to measure the percentage CD4 T cells. Thus, the updated version of the Auto40 flow cytometer, now based on a protocol using anti-CD45 and anti-CD4 monoclonal antibodies, is not only valid for CD4 T cell enumeration in absolute number, but also in percentage [21].

Due to the stability of its optical bench, the compact low-range single-platform Auto40 constitutes is a portable desk-top flow cytometer that can run on a 12-volt car battery and can be connected to a laptop computer. Thus, the Auto40 analyzer can be used in a mobile health care unit that could make CD4 T cell enumeration available in remote or hard-to-reach locations, as previously shown in a mobile health unit in Cameroon [22].

### Implementation of HIV monitoring laboratories for CD4 T enumeration by Auto40 flow cytometers in Chad

Chad is a large country with a surface area of 1,284,000 km^2^. This country is located within the Sahel, the horizontal strip of Africa lying between the Sahara Desert in the North and more fertile regions in the South. Its tropical climate has two distinct seasons: a wet season from May to September and a prolonged dry season that is marked by the harmattan, the hot (temperatures ranging between 22° to 45°C), and dusty winds from the Sahara Desert. Chad is characterized by a generalized HIV epidemic [25], with the adult HIV-1 sero-prevalence rate sitting at 3.5% in 2007 and reaching up to 8% in N’Djamena [1], and the HIV variants in these regions express high genetic diversity [26]. HIV prevalence in the South is 10% and in the East it is about 3%. UNAIDS reported that, at the end of 2010, 200,000 individuals from Chad were living with HIV infection, and 110,000 of those infected were women aged 15 years or older and 19,000 were children [1]. ART is indicated in greater than 55,000 individuals. Currently, it is estimated that only 6,500 people have access ART. The high level of antiretroviral drug resistance in patients on first-line therapy clearly raises the issue of adequate HIV laboratory monitoring in Chad [27].

**Figure 2:**
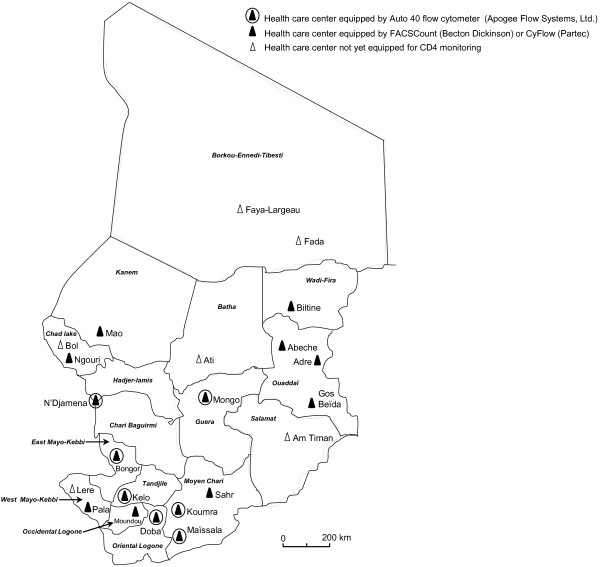
**Map showing the location of the 22 officially approved health care centers devoted to follow up adult and children patients infected with HIV-1 thorough Chad in 2013 (Am Timane, Abeche, Adre, Ati, Biltine, Bol, Bongor, Doba, Fada, Faya-Largeau, Gos Beïda, Kelo, Koumra, Lere, Maïssala, Mao, Mongo, Moundou, N’Djamena, Ngouri, Pala and Sahr), as well as the 8 centers equipped by the Auto40 flow cytometers (Apogee Flow Systems Ltd) (2 in N’Djamena at the hôpital Militaire D’Instruction, and the Centre D’Appui Psycho-Médico-Social Al-Nadjma; 1 in Mongo, Bongor, Doba, Kelo, Koumra and Maïssala), and the other 9 centers equipped by FACSCount (Becton Dickinson) (2 in N’Djamena; 1 in Abeche, Adre, Gos Beïda, Moundou, Pala and Sahr), or by CyFlow (Partec) (Adre, Biltine, Maïssala, Mao and Ngouri).** Note that 5 health care centers for HIV-1-infected patients (Ati; Bol; Fada; Faya-Largeau; Lere) are not yet equipped for CD4 T cell enumeration. The reference laboratory of the hôpital de la Liberté, N’Djamena, is equipped by FACSCalibur (Becton Dickinson). Note that only the health care centers involved in caring HIV-infected patients are shown in the map.

In 2008, the “Conseil National de Lutte contre le SIDA” in N’Djamena proposed an international tender for CD4 T cell analyzers to be used for HIV monitoring in antiretroviral treatment centers in Chad. Therefore, 8 Auto40 flow cytometers (Apogee Flow Systems Ltd) were acquired at the price of 23,500 € per analyzer for implementation throughout Chad. Two analyzers were installed in N’Djamena (at the hôpital Militaire d’Instruction, and at the Centre D’Appui Psycho-Médico-Social Al-Nadjma) and 1 analyzer was installed in HIV monitoring laboratories in Bongor, Doba, Kelo, Koumra, Maïssala and Mongo (Figure 2). Prior to installation of the analyzers at these sites, contractual training for the laboratory technicians and biologists was organized in June 2008 by the distributor of Auto40 flow cytometers in Africa (Inodia, Les Mureaux, France) at the hôpital de la Liberté in N’Djamena which was focused on routine use and preventive maintenance.

In addition, six CyFlow (CyFlow SL: 1; CyFlow Counter: 5; Partec, Muenster, Germany) were acquired at the price of 17,500 € in 2006 and were implemented in Abeche, Adre, Biltine, Koumra, Mao and N’Gouri. Five first-generation FACSCount (Becton Dickinson Biosciences, San Jose, California, USA) analyzers were acquired at the price of 37,000 € in 2003 and have been installed in N’Djamena, Goz-Beida, Moundou, Pala and Sahr.

### Evaluation of the routine usage of Auto40 flow cytometers in Chad (2008–2012)

The main characteristics examined to evaluate the performances of the 8 Auto40 flow cytometers distributed across Chad included: i) general characteristics of each laboratory (location, environment, and level); ii) provision of electricity and water; iii) availability of human resources; iv) reagents and consumables supplies; v) medical activity and CD4 T cell enumeration cost at each site; vi) quality assurance and control; vii) instrument support. In addition, an open questionnaire was performed at each laboratory focusing on the activity organization, the relations with the local technical support team and the distributor (Inodia, France), and more generally on all problems raised involving the use of Auto40 analyzers in Chad. The study was approved by the Chad Ministry of Public Health.

## Results

The results of the evaluation of the routine usage of the 8 Auto40 flow cytometers implemented in Chad for a period of 5 years (2008–2013) are shown in Table 1. The implementation of the Auto40 flow cytometers in the reference laboratory (level 1) of the hôpital Militaire d’Instruction, N’Djamena, in the district laboratories (level 2) of Bongor, Doba and Mongo, and in the health centers laboratories (level 3) of the Centre d’Appui Psycho-Médico-Social Al-Nadjma, N’Djamena, and of Kelo, Koumra and Maïssalis, was urban in N’Djamena and Doba, and rural in the other sites. Despite very high temperatures and the frequent presence of dust, analyzers could operate as non-contained units without air conditioning (except for two laboratories in N’Djamena and Doba) and with exposure to outdoor dust in Bongor, Kelo, Maïssana and Mongo, where the laboratories were ventilated only by ceiling and floor fans. Except for the hôpital Militaire d’Instruction in N’Djamena, where electricity works properly, power outages of electricity at the other sites were generally frequent. However, the frequent breaks of electric current in the majority of sites did not prevent the use of the Auto40 flow cytometer which uses electric generators as power substitutes, connecting the cytometer in series with an on-line UPS (Uninterruptible Power Supply). This latter device has a power supply of 2 KVA and a capacity of one hour, which was sufficient to maintain its use in the laboratory in cases of power outages. The use of sheath fluid recycling cassette proposed by the manufacturer for closed use of the Auto40 flow cytometer (as in a military vehicle) was chosen in all locations outside of the capital. Nevertheless, water was available in all laboratories where Auto40 cytometers were installed and there was the possibility of using locally-distilled water, in addition to the ability to deliver bottled distilled water to sites where the consumption and general laboratory activity are low. Furthermore, although transportable, none of the Auto40 instruments in Chad have been deployed on mobile units, Thus, the use of sheath fluid recycling cassettes obviously significantly increases the costs of consumables, since the cassette fluid recycling drive costs 921 € and it is recommended that the cassette fluid be changed after 1500 tests, are used in mobile health care units.

**Table 1:** Main characteristics describing the implementation and the 5 years usage (2008–2013) of 8 single-platform, volumetric, Auto40 flow cytometers for community-based point-of-care CD4 T lymphocytes enumeration installed in Chad

		**Location of Auto40 flow cytometers**							
**Catogories**	**Items**	**N’Djamena (hôpital Militaire D’Instruction)**	**N’Djamena (Centre D’Appui Psycho-Médico-Social Al-Nadjma)**	**Mongo**	**Bongor**	**Kelo**	**Doba**	**Maïssala**	**Koumra**
	Level of laboratory facility*	1	3	2	2	3	2	3	3
Laboratory characteristics	Situation	Urban	Urban	Rural	Rural	Rural	Urban	rural	Rural
	Distance from the capital city NDJ (km)	0	0	500	230	383	570	750	670
	Location in closed dust-free room (yes/no)	Yes	Yes	No	No	No	Yes	No	No
	Mean temperature gradient (minimum/maximum in °C)	22/45	22/45	22/45	20/40	20/40	20/40	20/40	22/40
	Air conditioned (yes/no)	Yes	No	No	No	No	Yes	No	
Electricity & water	Unreliable electricity power supplies	No	Yes	Yes	Yes	Yes	Yes	Yes	Yes
	Unreliable water supplies	No	No	No	No	No	No	No	No
Human resources	Biologist (number)	No	No	No	No	No	No	No	No
	Trained technician (number)	1	1	1	1	1	1	1	1
Reagents & consumable supplies	CD45, CD4 lyophilisated reagents	Yes	Yes	Yes	Yes	Yes	Yes	Yes	Yes
	Reagents delivery	Regular	Regular	Regular	Regular	Regular	Regular	Regular	Regular
	Reagents price (€)	10	10	12	11	11	12	12	12
	Sheath fluid recycling cassette	No	No	Yes	Yes	Yes	Yes	Yes	Yes
	Number of followed up patients (in 2012)	102	458	57	53	198	87	41	157
Medical activity in each site	Number of antiretroviral drugs-treated adult patients (in 2012)	95	421	55	49	137	82	33	145
	Number of antiretroviral drugs-treated children (< 5 years) patients (in 2012)	7	37	2	4	61	5	4	12
	Number of CD4 T cells enumeration** (in 2011)	341	1156	55	123	559	230	62	358
	Final cost of CD4 T cell count (in absolute number and percentage)***	11.0	11.0	13.0	12.0	12.1	13.1	13.4	13.2
Quality control	Assurance quality procedures	Yes	Yes	Yes	Yes	Yes	Yes	Yes	Yes
	Internal quality control	Yes	Yes	Yes	Yes	Yes	Yes	Yes	Yes
	International external quality control	No	No	No	No	No	No	No	No
Instrument Support	Routine maintenance on site	Yes	Yes	Yes	Yes	Yes	Yes	Yes	Yes
	Manufacturer’s maintenance (agreement)	Yes	Yes	Yes	Yes	Yes	Yes	Yes	Yes
	Troubleshooting	No	No	Yes (electronic memory card)	No	No	No	No	No

*Level 1: Reference laboratory; Level 2: District laboratory; Level 3: Health care center laboratory.

**Results of CD4 T cell enumeration with Auto40 flow cytometer are systematically given in absolute count as well as in percentage.

***The estimated final cost of 1 CD4 T cell enumeration does not take into account the cost of the use of sheath fluid recycling cassette in province laboratories, as well as the contractual maintenance of the Auto40 flow cytometers by the distributor (Inodia, France).

With regards to human resources, it is remarkable that users of Auto40 flow cytometers in Chad were exclusively senior laboratory technicians and there was an absence of biologist involvement in laboratory management at the survey sites. The reagents used in all sites included the combination of anti-CD45 and anti-CD4 monoclonal antibodies, thereby enabling for the enumeration of CD4 T lymphocyte absolute values and percentages, as the CD4 T lymphocyte count in percentage is only recommended for children between 24 and 59 months according to the 2010-revised WHO [2]. Lyophilized and heat-resistant reagents were preferred because of the high environmental temperatures, and the long distance of some sites from the capital. The supply of reagents was consistent and without disruptions. The estimated final cost for a CD4 T lymphocyte count in absolute value and in percentage was between 11.0 and 13.4 €. In Chad, this cost was estimated to be around 16 € for a CD4 T cell count given in absolute value and percentage obtained with FACSCalibur or FACSCount (Becton Dickinson), and 10 € with CyFlow (Partec).

The Auto40 flow cytometers were located in sites that supported mostly adults, with active files from 41 to 458 patients (mean of 144 patients per site), including a variable number of adults from 33 to 421 (mean of 127 per site) and a low number of children under 5 years from 2 to 61 (mean of 16 per site). In fact, the number of CD4 T cell enumeration per site was variable, with a mean of about 360 per year per location. However, some sites had very low activity, as in Mongo (n=55 in 2011) and Maïssala (n=62 in 2011).

A general procedure for quality assurance had been established in each laboratory by the National Executive Secretariat of the “Conseil National de Lutte contre le SIDA”, but this was not evaluated, mainly because the national network of laboratories was not yet operational in Chad. For each CD4 T cell count, the internal quality control procedures recommended by the manufacturer were performed, using calibrated beads (for Apogee Auto40-green). However, there was no registration of data collection of periodic internal quality control, as well as no temporal analysis of the obtained values, such as through the use of the Levy-Jennings approach, for example. Finally, no national or international external quality control for CD4 T cells enumeration, including CD4 monitoring by Auto40 flow cytometry platforms, were performed in Chad.

Technical support of the 8 Auto40 flow cytometers was organized as follows in Chad. There was no local distributor. However, a technician of the “Conseil National de Lutte contre le SIDA” overaw on-site national analyzers every 3 months (quarterly), and ensured dust removal, maintenance and periodic routine maintenance advocated by the manufacturer. In addition, a maintenance contract had been provided by Inodia (France), priced at 4500 € per Auto40 flow cytometer. In four years, such maintenance took place twice in 2011 and 2012. In the event of the occurrence of the most important technical problems to the analyzer, such as computer updating or breakdown, the design engineer of the Auto40 flow cytometer (from Apogee Flow Systems Ltd.) accompanied Inodia (France) in Chad at a price of 500 € per day. In total, it is possible to estimate the mean cost of the contractual maintenance performed by Inodia (France) at about 2300 € per analyzer and per year during the 5 years survey period. Note that all 5 FACSCount (Becton Dickinson) and 6 CyFlow (Partec) are also currently working properly in Chad. To our knowledge, a similar maintenance schedule has been organized in other Africa countries where Auto40 flow cytometers have been implemented [local distributor or technician; on-site maintenance organized by Inodia (France); specialized mission in case of unresolved issues organized by Apogee Flow Systems Ltd. (UK), concerning about 15 analyzers throughout sub-Saharan Africa.

In addition to the commercial service of reagents supply, and to on-site maintenance of the analyzers, Inodia (France) has been involved in 2012 in the national training of biologists and clinicians on the use in clinical practice of CD4 T lymphocytes count, in relationship with national health authorities.

## Discussion

In the present study, we addressed the principal issues raised over 5 years of routine usage of the Auto40 flow cytometers which had been implemented in 8 HIV monitoring laboratories throughout Chad. The installation and extended usage of a significant number of Auto40 flow cytometers in Chad also allowed us to assess the performance of these CD4 T cells counters in situations of actual use, across an entire country with harsh climatic conditions and a large geographical territory. This field approach is original, and complements the previous strictly biological evaluations of the analyzers in reference laboratories in Senegal [11], Cameroon [21,22], as well as recently in Chad [23].

The analyzers were able to function properly regardless of the level of the laboratory, including in level-3 laboratories in health centers. They were even able to function properly in rooms partially exposed to the outside environment, which were not air conditioned, which had widespread dust, and in settings where there were frequent electricity outages. In each site where an Auto40 flow cytometer was located, a senior laboratory technician was able to proficiently replace a biologist, at least for counting CD4 T cells with standardized reagents and using a simple protocol. Of major interest is our finding that the A40 technology may be easily implemented and maintained by technicians. Indeed, well-trained technicians were able to use the Auto40 flow cytometer with low intra- and inter- run variability of less than 10% [21,22]. This value was comparable to other published reports using single-platform flow cytometers [28,29], and is considered acceptable in routine clinical practice [29]. A single failure of an electronic nature occurred in only one analyzer during 5 years of use. Routine maintenance recommended by the manufacturer was performed regularly, as was the contractual annual maintenance by the technician of the distributor arriving from France. Altogether, these observations of actual use in tropical conditions confirm the reliability and robustness of the Auto40 flow cytometer, originally designed for military applications [11,21,22].

One of the interesting characteristic features of the Auto40 analyzer is its use of stabilized monoclonal antibodies. The assay reagents can be stored for prolonged periods of time (up to 12 months) at high temperature without any loss of biological activity [30]. The use of thermo-resistant reagents allowed reliable measurements of CD4 T cell count, both in terms of absolute numbers and percentages, under unfavorable conditions such as high temperatures encountered in the tropics and in remote areas where cold conditions for the storage of reagents, particularly during shipment and delivery, are not guaranteed [31]. The thermo-stable monoclonal antibodies used in the present study can be kept for as long as one year at room temperature (30°C), and have been chosen for all laboratories equipped by an Auto40 flow cytometer in Chad. The possibility of long-term storage of reagents at room temperature for one year should facilitate the planning of laboratory activities and reduce the costs related to loss of reagent stability. Overall, the use of thermo-stable reagents increases the accessibility to flow cytometry testing. However, while the manufacturing costs related to the antibody stabilization procedure does not exceed 15% of the original cost [30], the final sale prices of thermo-resistant reagents marketed by Inodia (France) is higher than 40% compared to those of liquid reagents. In practice, cost reduction on reagents for CD4 T cell counting could only materialize with active pressure on manufacturers and likely their agencies, in order to reduce production, administrative and logistical expenditures acting to inflate the costs of laboratory intrants in resource-limited settings.

Some criticisms which do not *stricto sensu* address the intrinsic qualities of the Auto40 flow cytometer can nevertheless be made based on the use of the analyzers in Chad. First, the price of the Auto40 flow cytometer is about 30% higher than its principal competitors sold in Chad. The choice of reagents is also somewhat problematic. Indeed, if the use of heat-stable reagents is indicated in Chad, the choice of using monoclonal anti-CD4 and anti-CD45 antibodies as reagents to systematically render the number of CD4 T cells in both absolute number and percentage appears unfounded. Indeed, given the percentage is only recommended in children between 24 and 59 months, its measurement may not be of interest in individuals over the age of five years [4]. The choice of routine use of anti-CD4 and anti-CD45 antibodies doubles the cost of CD4 T lymphocyte counting. As the number of children represents less than 15% of followed patients, it may have been possible in most treatment centers where the number of treated children is very low to enumerate CD4 T cell absolute numbers exclusively, since the treatment initiation threshold of 750 CD4 T cells/μl is now recommended by WHO in children between 24 and 59 months [4,5].

The distribution of Auto40 flow cytometers was also questionable, given the very low clinical activity at some sites. Half of the 8 cytometers were installed in sites where the number of CD4 T measurements performed was less than 300 per year, despite the fact that each cytometer can perform 150 tests per day. Only two Auto40 flow cytometers (in N’Djamena and Kelo) had significant usage, performing over 500 tests a year. Moreover, the systematic use of sheath fluid recycling cassettes in provintial sites appears to have little to no justification as it results in an obvious increase in cost. Considering the relatively high costs involved (Auto40 flow cytometers themselves, reagents, sheath fluid recycling cassettes, contractual maintenance), and the low to very low medical activity in most sites, it appears that the cost of CD4 T cell enumeration using Auto40 flow cytometers may be excessive for some sites.

These observations raise the issue of decentralization of immunological monitoring in large areas where laboratories are remote. One solution could be to use mobile health units capable of performing immunological monitoring of HIV-infected patients [32], as shown recently in Cameroon [22]. Another option could be to send blood samples, possibly with a blood preservative [33], to a reference or district laboratory equipped with a CD4 T cell counter. The lack of external quality control programmers, such as those proposed for resource-limited settings by QASI [34,35] or AFREQAS [36], is another flaw and likely stems from the lack of operational national network of laboratories in Chad.

At the present time, the northern distributor (Inodia, France) has installed only a few (less than 20) Auto40 flow cytometers in sub-Saharan Africa. Thus, the Auto40 flow cytometers implemented in Chad represent more than one-third of the analyzers installed in Africa by Inodia. This represents, therefore, a significant business favored by the northern distributor who is partly responsible for: a) implementing flow cytometers in peripheral laboratories of health care centers with a very low activity; b) choosing the most expensive reagents poorly adapted for routine use in some sites; and lastly c) using expensive sheath fluid recycling cassettes in the provincial laboratories to overcome the problems of disposing clean water. These cassettes have been designed for autonomous usage in Auto40 flow cytometer in military vehicles (in case of biological war), and appear oversized and ill-adapted in the context of Chad. Indeed, it is always possible to supply the provinces with bottled distilled water or to produce it locally. It would be catastrophic for Chad if the distributor or manufacturer declared bankruptcy because the resulting interruption of reagents delivery and maintenance of Auto40 cytometers.

Collectively, the Auto40 flow cytometer, which originally had been designed for military use, is actually a reliable and robust analyzer. The version conceived for CD4 T cells counting in resource-limited settings provides correct and reproducible results, in terms of both absolute values and percentages. However, the costs of the cytometer and the consumables (notably sheath fluid recycling cassettes) and the contractual maintenance are elevated. The high functional capacity of the cytometer (150 samples per day), as well as its possibility for open use for several types of analyses other than the quantification of CD4 T cells, is possible in only a limited number of reference laboratories. Compared to other systems of CD4 T cells counting designed for resource-limited countries [9], it appears that the Auto40 flow cytometer is most suitable for use in reference laboratories (level 1) and possibly in district laboratories (level 2) if their clinical volume is sufficient. Other systems of CD4 T cells counting, such as the recently proposed POC systems [9], should be selected in remote laboratories like those of many health care centers. Another use for the Auto40 flow cytometer could be the enumeration of CD4 T cells within mobile therapeutic units [22]. However, although the mobile unit concept of HIV infection diagnosis and treatment involving community health workers would be particularly adapted for isolated populations living in remote villages, as previously shown in Cameroon [32], the Chad health care system has not yet been capable of implementing a coherent program of “mobile” health care for decentralization. Finally, the Auto40 analyzer could be a reference flow cytometer allowing external quality control, thus fulfilling some of the objectives of the national laboratory network.

## Conclusion

In conclusion, the simplified, single-platform, volumetric Auto40 flow cytometer is a reliable alternative flow cytometer for CD4 T lymphocyte enumeration which can be used in routine immunological monitoring in resource-constrained settings like in Chad. In addition, our observations suggest that a CD4 T cell analyzer should be chosen carefully according to the needs of each care site, and the choice of analyzer should take into account the environmental conditions and the necessity for decentralization. Thus, an Auto40 mini flow cytometer is not suitable for all laboratories as it is clearly oversized and ultimately very expensive. According to our field experience and the present evaluation, we recommend its usage mainly in reference laboratories (level 1) and in district laboratories (level 2) if the clinical activities at these sites are sufficient, but not in laboratories of health care centres (level 3). As there is now a large selection of CD4 T cell counters for resource-limited countries, the equipment needs for CD4 T cell analyzers nationwide must be clear to several manufacturers so that they are encouraged to offer complementary materials adapted to a limited number (2 to 3) of health care sites.

## Competing interests

The authors declared that they have no competing interests.

## Authors’ contributions

DK and LB conceived and designed the research and analyzed the results. DK, LB, MAJ and ANM drafted the manuscript; BD was directly involved in the implementation of Auto40 analyzers in Chad; NDO discussed the medical validity of CD4 T counting by the Auto40 analyzer; DK, MAJ, FXMB, ANM and LB addressed the issues raised by the referees. All authors read and approved the final manuscript.

## Pre-publication history

The pre-publication history for this paper can be accessed here:

http://www.biomedcentral.com/1472-6963/13/373/prepub
